# Model for developing context-sensitive responses to vulnerability in research: managing ethical dilemmas faced by frontline research staff in Kenya

**DOI:** 10.1136/bmjgh-2021-004937

**Published:** 2021-07-08

**Authors:** Sassy Molyneux, Priya Sukhtankar, Johnstone Thitiri, Rita Njeru, Kui Muraya, Gladys Sanga, Judd L Walson, James Berkley, Maureen Kelley, Vicki Marsh

**Affiliations:** 1 KEMRI-Wellcome Research Programme, Centre for Geographic Medicine Research Coast, Kilifi, Kenya; 2 Centre for Tropical Medicine and Global Health, Nuffield Department of Medicine, University of Oxford, Oxford, UK; 3 Department of Child Health, Gloucester Hospitals NHS Foundation Trust, Gloucester, UK; 4 Ethox Centre and Wellcome Centre for Ethics & Humanities, Nuffield Department of Population Health, University of Oxford, Oxford, UK; 5 Kemri-Wellcome Trust, Centre for Geographic Medicine Research Coast, Nairobi, Kenya; 6 Department of Global Health, University of Washington, Seattle, Washington, USA

**Keywords:** child health, hospital-based study, paediatrics, public health

## Abstract

Health research in low-resource settings often involves individuals and populations defined as ‘vulnerable’. There is growing attention in the literature to the ethical dilemmas that frontline research staff face while conducting such research. However, there is little documented as to how research staff might support one another in identifying and handling these dilemmas in different contexts. Over the course of conducting empirical ethics research embedded in the Childhood Acute Illness & Nutrition Network, we developed an approach to examine and respond to the ethical issues and dilemmas faced by the study teams, particularly frontline staff. In this paper we describe the specific tools and approach we developed, which centred on regular cross-team ethics reflection sessions, and share lessons learnt. We suggest that all studies involving potentially vulnerable participants should incorporate activities and processes to support frontline staff in identifying, reflecting on and responding to ethical dilemmas, throughout studies. We outline the resources needed to do this and share piloted tools for further adaptation and evaluation. Such initiatives should complement and feed into—and certainly not in any way replace or substitute for—strong institutional ethics review, safeguarding and health and safety policies and processes, as well broader staff training and career support initiatives.

**Key questions**

**What’s already known?**

- Health research in low-resource settings often involves individuals and populations defined as ‘vulnerable’.
- There is growing attention in the literature to the ethical dilemmas that frontline research staff face while conducting research involving vulnerable individuals and populations, but there is little documented as to how research staff might be supported in identifying and handling these ethical dilemmas post ethics approval.

**What are the new findings?**

- We describe the specific tools and approach developed to support frontline staff in working through ethics issues faced over the course of conducting research in contexts of vulnerability.
- The approach we share centres on regular cross-team ethics reflection sessions, which overlap with structured debriefing and reflexive practice in qualitative research, and hospital ethics support processes in clinical care.

**What do the new findings imply?**

- All studies/trials involving potentially vulnerable participants should incorporate activities and processes to support frontline staff in identifying, reflecting on and responding to ethical dilemmas.
- Such initiatives must complement and feed into (and not compensate for or undermine) strong institutional ethics review, safeguarding and health and safety policies and procedures, as well as broader staff training and career support initiatives.

## Introduction

High-quality ethical health research is essential to tackle disease burdens in low-resource settings.^1 2^ Within low-resource settings, disease burdens are disproportionately shouldered by communities, families and individuals defined as ‘vulnerable’ in health research ethics guidance.^3 4^ People and groups defined as vulnerable in health research are those who—by virtue of their circumstance, or physical, social, political, or psychological conditions—are especially susceptible to harms, ill health and coercion or exploitation by others.^5 6^ They are also especially in need of healthcare that has an evidence-base relevant to them. A central challenge at the heart of planning and reviewing research involving vulnerable populations is a paradox that overprotection can block needed research, while research without adequate support and benefits can worsen vulnerabilities. In conducting ethical health research, there is the challenging task not only of recognising and responding to multiple potential layers of vulnerability but also of understanding, protecting and building on people’s agency.^7–9^

Given the ongoing high morbidity and mortality among young children in low-resource settings, there is a particular need for paediatric clinical research that includes young and underprivileged children, their families and communities.^1 2^ As a recognised vulnerable group in research ethics guidance, children require particular justification for their inclusion in clinical research in relation to the direct benefits they receive and the potential benefit for future populations facing similar illnesses.^5 6^ There is a relatively high bar set for research involving children with regard to assessing risk thresholds over ‘minimal’ levels (eg, risks associated with daily living)^5 6^ and requirements for special protections incorporated through consent and assent processes and ancillary care and benefit-sharing plans.^4 10^

Although guidance to support ethical clinical paediatric research is in place, it is far from straightforward to apply in practice, even for research with widely agreed social value. Consent processes are highly challenging to administer on the ground,^11–13^ with particular difficulties for facility-based research where there may be blurring between research activities and clinical care and where children’s fathers—often important decision-makers in households—may not be present.^14^ For ancillary care, contributing adequate support and benefits to participants and their family members has to be weighed against risks of undue inducement for families to participate and guardians essentially being offered ‘an empty choice’.^15 16^ Further potential challenges with relatively high levels of benefits include: (1) introducing relationship challenges between participants and non-participants in the same health facilities or communities, as a result of perceived unfairness in who is receiving what and (2) participants receiving care or items that make them different to typical patients, which in turn undermines the generalisability of the study findings to other settings.^16 17^ While many such ethical issues are considered and addressed in advance through research proposals and plans, associated ethical challenges and dilemmas inevitably still emerge post approval.

Researchers at all levels report needing better guidance and support for working with vulnerable participants and their families in order to better respond to needs and ethical dilemmas.^17–20^ Many of the ethical challenges and dilemmas that emerge post approval are faced by frontline research staff, with challenges emerging either as dramatic and extreme cases, or as more routine feelings of discomfort in everyday research practice. As Guillemin and Gillam highlight, giving credence to moments of discomfort is ethically important a crucial part of building up our ‘ethical mindfulness’^21 22^ or our ‘everyday’ ethics practice. Although there is a growing literature on the ethical dilemmas that frontline research staff—particularly non-medically trained ‘fieldworkers’—face,^18 19 23 24^ there is relatively little documented as to how research staff might support one another in identifying and handling these dilemmas as they arise. Frontline research staff therefore often struggle on their own in working out how best to balance these ethical considerations in daily research practice.^18 19 24 25^

One approach to providing support to frontline staff with ethics issues that arise post approval is through participatory training. Such training can include discussing the anticipated issues and agreeing their appropriate handling.^24 26^ While important, this training does not necessarily offer on-going structured support as issues arise. Another approach is to build support into institutional safeguarding initiatives, which are increasingly required for organisations working with vulnerable groups.^27^ The United Kingdom Collaborative on Development Research (UKCDR) has defined safeguarding in international development research as preventing and addressing ‘any sexual exploitation, abuse or harassment of research participants, communities and research staff, plus any broader forms of violence, exploitation and abuse… such as bullying, psychological abuse and physical violence’ (cited in Aktar *et al*^27^). While safeguarding issues may overlap with ethical concerns for frontline staff and be similarly related to vulnerabilities caused by unequal power relationships (between staff and between staff and participants/community members), not all ethical issues that frontline research staff face are safeguarding issues and vice versa. Furthermore, the literature on safeguarding in global health research is itself very limited.^27^

In this paper, we draw on work conducted in Kenya as part of a wider empirical ethics study embedded within a multisite, multidisciplinary clinical observational study.^28^ The main aims are to: (1) describe the approach we developed to help identify, unpack and respond to the ethical issues and dilemmas faced by frontline staff in their interactions with research participants facing multiple, layered vulnerabilities and (2) share our learning from using this approach, including in relation to the ethics issues faced and how they were categorised and responded to. The empirical ethics work was part of an international study entitled ‘Resilience, Empowerment and Advocacy in Women’s and Children’s Health Research’ (REACH). The overall aim of the REACH collaboration is to contribute to a more nuanced understanding of vulnerability in research ethics and improved practical ethical support and guidance for ethically responsible research. The work includes empirical ethics case studies in Kenya, South Africa and Thailand.

## Methods

As REACH researchers, we conducted embedded ethics and social science research linked to the Childhood Acute Illness & Nutrition (CHAIN) Network observational cohort study^29^ in two Kenyan sites: Kilifi and Nairobi.^28 30^

### The REACH study and the CHAIN Network cohort study

**Figure 1:**
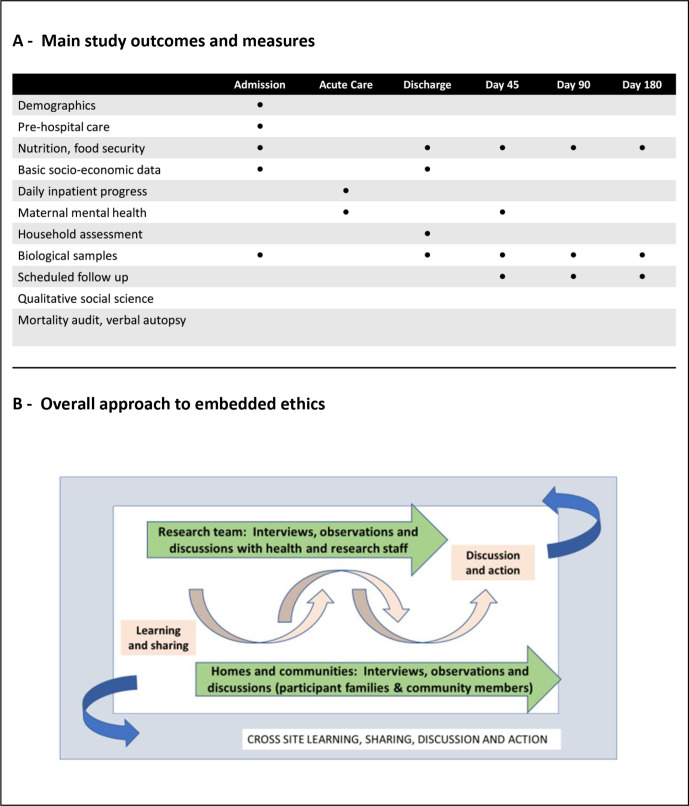
Childhood Acute Illness & Nutrition Network outcomes, measures and the embedded ethics approach.

CHAIN (www.chainnetwork.org) is a multidisciplinary research network aiming to understand the mechanisms contributing to young child mortality in hospital and after discharge in Low and Middle Income Countries (LMICs) in order to identify interventions to improve survival.^29^ The Network conducted a prospective observational cohort study at nine hospital sites in Africa and South Asia, recruiting more than 3000 acutely ill children at admission to hospital and following them for 6 months after discharge to identify pathways underlying mortality risk despite adherence to current treatment guidelines and protocols. The main study procedures in CHAIN are outlined in figure 1 (top image), with the overall design being observational, that is, building into, learning about and referring to existing services rather than creating new systems for study participants.

The integrated REACH empirical ethics study included input on overall study design, consent processes, ancillary care planning and regular review and discussion of emerging ethical issues and dilemmas across all sites (MK served as an ethics advisor for the CHAIN Network). Cycles of learning, advice, discussion and corresponding action were embedded into study processes, including annual meetings, as outlined in figure 1 (bottom image). These cross-site processes were informed by in-depth qualitative research involving research team members and homes and communities in four sites, including Kilifi and Nairobi counties in Kenya.^31^

### Study setting and ethical context of the research

The Kilifi and Nairobi research sites in which we conducted qualitative research are linked to the Kenya Medical Research Institute (KEMRI)-Wellcome Trust Research Programme (KWTRP), which is a multidisciplinary, internationally recognised health research programme with a long history of community engagement and empirical ethics research. Deliberative processes including community members have contributed to KWTRP guidance for informed consent and benefit-sharing and data-sharing processes.^16 32–34^

Kilifi is one of the poorest counties in Kenya, with 68% of the population living below the poverty line. Most of the population depend on small-scale farming, and high levels of gender inequity are documented.^35–37^ Kilifi County Hospital has a robust collaboration with KWTRP. In Nairobi, research was conducted in two large informal settlements (Kibera and Mathare). It is estimated that 56% of residents in Nairobi live in informal settlements, which are characterised by poverty, high burdens of disease and mortality, limited access to healthcare, severe financial constraints, inadequate access to water and good sanitation and poor housing conditions and livelihood opportunities.^38–40^

As part of CHAIN, we explored children and family members’ vulnerability and agency across treatment-seeking journeys and research encounters, drawing on data gathered from repeated interviews and observations with family members of 20 and 22 children purposively recruited from the primary CHAIN cohorts in Kilifi and Nairobi, respectively (total n=42 children). The vulnerabilities family members faced, their ‘journey’ through the health system, the agency revealed in their stories and the way in which research encounters were woven throughout have been published elsewhere,^31 41 42^ with the findings summarised in the online supplemental files 1 and 2.

10.1136/bmjgh-2021-004937.supp1 Supplementary data

10.1136/bmjgh-2021-004937.supp2 Supplementary data

Families’ stories reveal the context within which family–research staff interactions were taking place. Although family livelihoods varied, many faced low, irregular sources of income, competing demands on those resources, complex and dynamic family situations (such as physical separation, regular movement or divorce) and gendered, sometimes challenging, family and community relations. By the time children reached hospital, many carers had already undergone complex and lengthy treatment-seeking journeys, often experiencing significant challenges linked to the multilayered situational vulnerabilities and health service constraints they faced. Carers’ persistence in the face of these extremely difficult challenges demonstrated agency, but this agency was shaped and significantly constrained by structural drivers beyond their control, such as scarce income-earning opportunities, seasonal drought and food shortages, poor access to quality facilities and norms around who should make decisions in families. These contexts inevitably influenced family members’ hopes, expectations, fears and concerns regarding the research, in turn shaping the ethical dilemmas that frontline research staff faced in conducting the study.

### Data collection and analysis on ethics issues faced and responses

In this paper, we draw on three inter-related sets of qualitative data collected by social scientist teams in Kenya.

### Formal interviews with frontline staff

We conducted formal individual interviews with five frontline staff (three clinicians and two fieldworkers) who worked closely with CHAIN participants to understand their perspectives on children’s illness trajectories and family influences, any ethical dilemmas they had faced in conducting the CHAIN and related research and about how they handled such dilemmas. These individual interviews, supplemented by observations and informal discussions with a diverse range of frontline staff, fed rapidly into the establishment of the ethics reflection sessions described below. Data from formal interviews were entered into Nvivo V.10 as a separate data set and analysed using a framework analysis approach.

### Social science team meetings

Second, we have drawn in this paper on notes from our regular social science group meetings where we began to share the ethical dilemmas we were experiencing as a social science team as a result of what we were being told by household members and staff and what we were seeing. To prepare this paper, we extracted data from the detailed minutes of those meetings (n=15 sets of notes) across three themes: the dilemmas we faced in detail, discussion and debates on our responsibilities to act and the reasoning and any agreed actions. Among the issues we faced and discussed were frontline CHAIN staff beginning to raise issues informally or indirectly with us with comments such as ‘I’m not sure what I should do when…’ or ‘when do I know if I have done enough?' Often, we would be told ‘I have to confess that’ or ‘off the record…’ and sometimes we would hear, usually in jest, that we were considered ‘the FBI’ or the ‘ethics police’. This highlighted the need to work sensitively within existing research hierarchies and to build trust over time.

In response, we developed three inter-related tools and approaches to integrate ethics discussion into CHAIN. First, we implemented a communication skills training course tailored to CHAIN frontline staff drawing on course methods and materials regularly used across the research institution and which recognise a core role for emotions.^26 34 43^ The course included discussion and role plays on experienced and anticipated challenges and dilemmas for research staff in communicating with study participants. A training report crosschecked by all participants before being shared with senior CHAIN staff included issues raised and recommendations drawn on in ethics reflection sessions. Second, we introduced personal diaries to prompt and support frontline staff to identify, document and share any ethics dilemmas faced in their daily work (ie, where they were not quite sure what the right thing to do was or felt unable to do what they felt was right). In Kilifi, diaries were physical books, which staff could later draw on to type or write anonymously into a word document and, where this was comfortable, hand in to an administrative assistant in advance of the ethics reflection meetings. In Nairobi, diaries were electronic documents. Following an initial flurry of submissions, we phased this activity out as issues began to be freely and spontaneously raised in ethics reflection sessions as the practice became more familiar.

### Ethics reflection meetings for CHAIN frontline staff

Third, and drawing on earlier experience, we evolved a series of ethics reflection sessions to provide a safe space for frontline staff in CHAIN (clinicians and fieldworkers) to raise and discuss the ethics dilemmas they were facing with colleagues and supervisors and responsibilities for action. Sessions lasting 2–4 hours involved all CHAIN team members, including the site Principal Investigators (PIs, although not always), coordinators, frontline staff, social scientists and external ethics expertise (MK) (total n=8 meetings). These monthly meetings were separate from weekly business-like CHAIN meetings, which had a prior agenda and progress updates. Reflection meetings had a more open agenda and informal feel, including tea and snacks. Meetings were not recorded to protect a sense of a shared safe space but this paper draws heavily on the detailed notes taken by allocated note takers. These notes were reviewed and approved by all team members at the start of the next meeting.

As part of the ethics reflection discussions, we evolved a tool to help us more fully understand each situation being described and to identify and explore ethical issues and researcher responsibilities. This tool drew heavily on the benefit-sharing and ancillary care literature and particularly Richardson and Belsky’s partial entrustment model^44^ where researchers’ responsibilities to participants (and families) are linked to: the type and extent of participant vulnerability; their level of dependency on researchers to provide the benefits; the intensity/duration of researchers-participant relationships; researchers’ gratitude (for uncompensated burdens or costs participants have incurred); the impact of acting on the science and the moral costs of mobilising resources.

**Figure 2:**
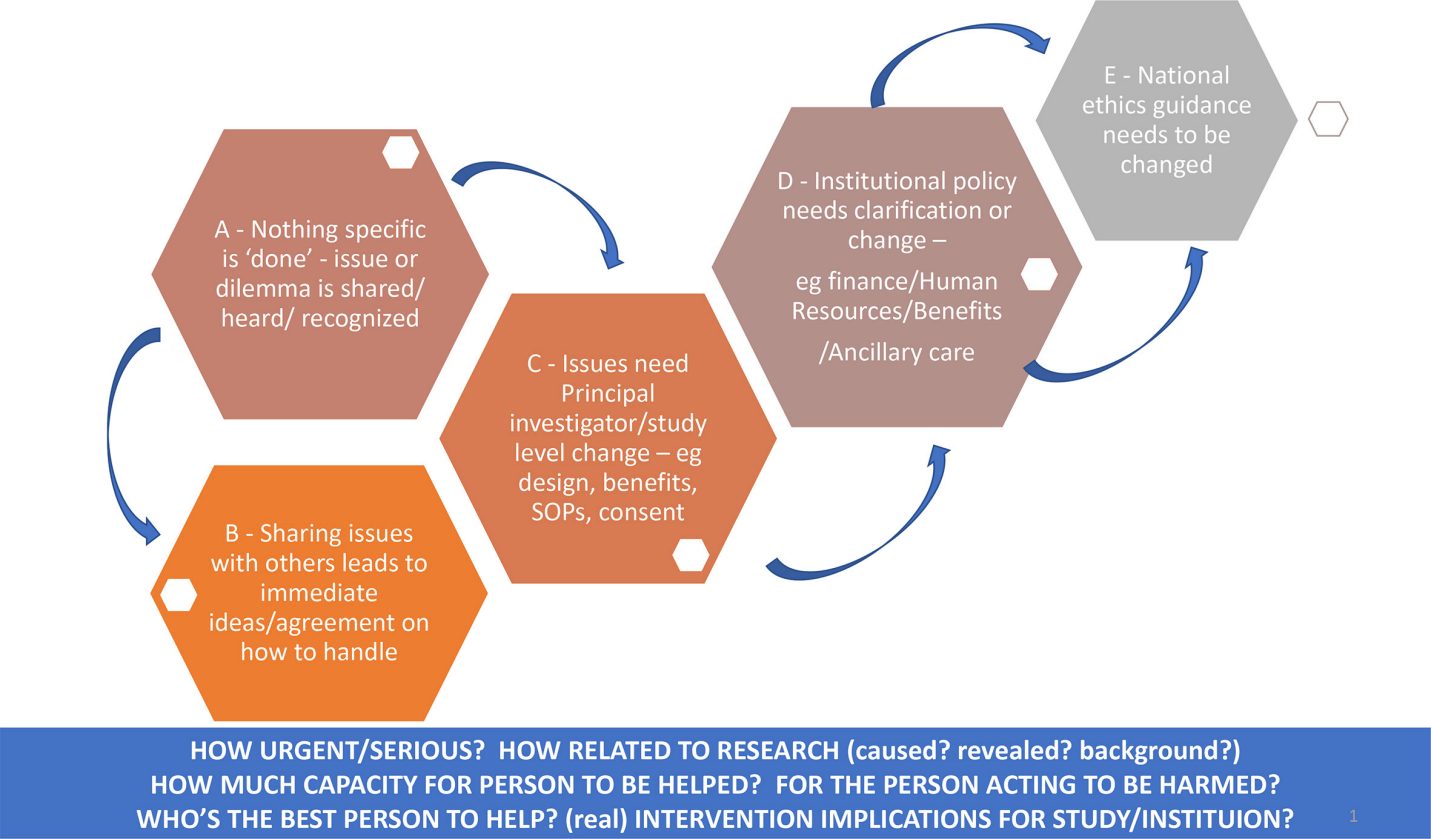
Tool to support discussion on researcher responsibilities and illustrations of how we categorised some of our dilemmas.

The different potential types and levels of action to consider are illustrated in the hexagons A–E in figure 2: in level A, there may be no action required or possible, but at least for the staff members sharing the dilemmas, they had an opportunity to share the dilemma and have it acknowledged and know whether others have faced similar concerns. In level B, there may be an immediate action necessary or possible, through an agreed, possibly better, way of acting or communicating in this and similar situations. This might require level C: which is some change across the trial team through, for example, changing the study design, SOPs, consent processes or benefit packages. Such changes may well require a protocol amendment submitted to and approved by national and international ethics committees, as well as amendments to institutional guidance on, for example, benefit-sharing or consent policies (level D) and possibly even recommendations for national ethics guidance changes (level E).

### Patient and public involvement

This research focuses on the ethics-related dilemmas and support processes of frontline staff rather than patients or the public. The paper draws on data from research participants, including hospital users (see also^25 35 36^), and on insights provided by research participants and the public in previous engagement activities on related topics in the area.^15 18 28 37^ However, we did not include patients or the public in the design, conduct, reporting or dissemination plans of the data presented in this paper. We are currently planning a video to share the findings and implications with a wide range of stakeholders.

## Results

We provide an overview of the range of ethical issues faced by frontline staff, followed by some examples of our agreed responsibilities and actions in relation to the issues raised and the lessons learnt. In the discussion, we relate our approach to the literature and consider the wider implications.

### An overview of the ethical issues raised by frontline clinical and non-clinical staff

Issues raised across the above mechanisms could be broadly grouped into those related to: (1) consenting, (2) conducting study procedures, (3) difficulties in disentangling the personal and the professional and (4) ending the study (table 1). In this short paper, we cannot do justice to the range and complexity of the various dilemmas shared by frontline staff, but some illustrations follow.

**Table 1:** Ethics issues and dilemmas raised by frontline staff

Consenting for and ending the study	Conducting study procedures: blood sampling, compensation and ancillary care	Broader interactions and relationships
Initial consent processes Having to seek either full consent or nothing—no assent process Parents not wanting all of the information or not listening Parents put off by blood samples What to do where a mother consents and then someone else wants her to change her mind—what if the mother still wants the child to be in the study Consenting controls How can we ask them to give samples when their children are not even sick? Ending the study Families wanting to stay part of KEMRI or believing the child must be well Ending the study when a child has died and verbal autopsies (VAs) Are some families who lose study children being subjected to two VA Feeling underprepared and undersupported to conduct VAs	Some elements of the study emotionally challenging Blood sampling: Painful to hear children cry and mothers upset Socioeconomic status, nutrition and mental health questions: Because of the questions that have to be asked, getting to hear about, for example, how hungry families are and about high levels of depression Levels of compensation of families: ‘Small’ differences between studies in what is given/paid for a routine study clinic—for example, if a banana is given, and 350/=vs 300/= Lack of clarity on lunch provision for hospital visits over lunch and whether food/fares can be given to others accompanying the mother to hospital Home visits for research costing household members money (cannot earn) or preventing household (HH) tasks, especially where take longer than expected Why cannot we give more to people who earn more, otherwise they will drop out? Referral of inadequate perceived quality or unlikely to be taken up Where the types of clinical needs are way beyond what’s easily desirable, available or affordable to families Where does a referral end? For example, depressed mother	Ability to help as fellow community members What we are allowed to give/do as we normally would as clinicians or as community members without undermining the study procedures and goals? Attending funerals Can/should field staff attend funerals where children have died in studies? Can they offer a condolence fee? Levels of compensation of staff for emergency costs incurred Being unable to refunded by the research centre for unexpected costs incurred out of humanitarian support Other Handling romantic advances from study parents to staff

### Consenting for the study

We noted above the emotional context of admission to hospital (the point of recruitment into the CHAIN cohort), which was also when most consent processes took place. Family members had often visited multiple healthcare providers and incurred significant financial burdens (and sometimes insulting and humiliating encounters) when seeking care for their child.

I was tired and there is no hospital I had not been to, I had visited several hospitals, wasn’t I from another hospital when I came there? So, I had to accept so that I may know what the problem is… Mother.Hh.6, Rural (see ref 41, p9).

I had no hope, I had lost hope, and my brain couldn’t take in anything. I had lost hope… I was confused. Mother. Hh3, Rural (see ref 41, p7).

The consent process itself, including emphasising voluntariness and the need to make a choice, amplified the emotional context of admission, sometimes adding to caregivers’ worries and frustrations and sometimes feeding into more positive emotions of relief, hope and trust.^41^ For frontline health staff, corresponding dilemmas included whether the information they gave was heard and understood (box 1, quote 1).

Box 1 **Illustrations of ethics issues faced**

**Recruitment and ending the study**

1 - Sometimes you give a parent a consent and then at the end of the day the way they give you the feedback or the way they respond you ask yourself ‘Did this parent really understand the consent? Did she understand this is just a research….(that) she can either choose to participate or not? Cause you gave somebody a consent and then at the end she tells you ‘its ok because I just want my child to get better’. You see so you are left wondering should I consider this refusal or is this acceptance? (clinical frontline staff, 005)

2 - If by any chance this child—maybe because of the ongoing clinical problem—deteriorates… I have had quite a number and even probably my colleagues would testify to this, (that) usually the first blame is research as a cause of the deterioration. Yeah even if you go and revise like this is just the ongoing clinical problem that the child had going on but for them they will be attached (to the idea) that ‘my child has been bled when admitted; the child has been bled again right now; my child has deteriorated …(clinical frontline staff, 004)

3 - You tell the parent we will do our best… our clinicians will do their best to make the baby get good treatment. But then after two or three days the child dies, and they come to you and tell you ‘mtoto wenu amekufa, mtoto wenu amekufa’ (your child has died, your child has died), now they say mtoto wenu ((P: Your child, your child)) your child has died, you don’t know how to… I think you’ll just find a way, you find somewhere to hide first, (but) then you will come later, so it becomes challenging. Like you are the one who normally visits them on the bed(side) every day to check on them just to create a good rapport with them, so if something goes wrong, you are supposed to be there to console them, and what are you going to do? You can’t even pay for their bill, you can’t even give them consolation money just to carry the baby home, you can’t. You are just there the study has ended for them, just like that (fieldworker, 002).

**Research procedures**

4 - (We hear about difficult situations) many, many times: I’ll give an example… I was admitting a child yesterday and this child has not seen their mum for four months… From the history given by the grandmother who brought the child this mother had separated from the husband. And this is a family who at most they are getting one meal in a day, and there’s actually no, no direct source of income that is attached to that particular family. It’s like sometimes they have to borrow from the neighboring homesteads, sometimes they need to just wait and make some funny herbs (wild vegetables) which are around so that they can make a meal. So, I think it’s, it’s, if you sit there with a patient and discuss about that, sometimes you feel, you feel, regret… why did I ask something that I can’t, I can’t help now? (clinical researcher, 005)

5 - (Referring to a household visit) Since you feel touched by her case you can just decide to chip in and help. Sometimes you even regret asking those questions, ‘why am I asking these questions yet by the time I am done I will not have helped in any way’ but this is work it needs the answers but by the end of the day how will you have helped? Like a question may ask, ‘in the past week has anyone gone to bed hungry?’ she says ‘aaah that’s the norm!’. At the end you leave her like that, she tells you ‘we haven’t eaten anything since yesterday up to now’ then you are like ‘ok mother I am leaving’ you switch on your land cruiser and leave but you feel like, why was I asking these questions yet I could not chip in even if it’s saving the situation today and tomorrow? (fieldworker, 003)

6 -In the parental health questionnaire called the PHQ9, if the mother is stressed, that is if she has a high score, we refer her to a social worker who counsels her and sees how they can help her. But there are some who have not scored the percentage CHAIN requires and you can just see that this one has a problem but is trying to maintain (hold themselves together)….(She falls below our cut-off for referral which)… means she is still stressed but we cannot refer her; we can only reassure her or ask the nurse to talk to her (clinical researcher, 005).

7 - Ah (I’ve helped out) many times cause … you’ll see mothers in the ward… they have been discharged in the ward and they have stayed there for almost let’s say three days, they are only waiting for a Friday where the waiver committee sits to decide whose bill is waived. At the same time this mother, because now you are in wards everyday doing the wards round, they have opened to you a lot of unsettled issues and you realize they tell you even if we get discharged here I am not seeing anything good, probably I am likely to come back cause you will discharge me here and I don’t have food to eat so where do I go? And I have other children that the mother left me with that I should go and take care of. Sometimes you feel like what will it harm when I give this mother a thousand shill…. a thousand bob? What harm will it have? And then it’s not because I am so philanthropic but the feeling that I am leaving you without any food to eat knowing you told me that you have nothing to eat…. sometimes it’s human you just see yourself going to just give it (clinical researcher, 005)

In response, staff often highlighted the specific information they felt parents wanted and needed, rather than covering all elements of the consent form. Taking parents’ time was especially a concern where children were very sick (noting that children requiring emergency cardiopulmonary resuscitation were excluded from the study as laid out in the research protocol). While recognising the importance of including these children in the study, staff queried the need for a full informed consent process on recruitment, preferring initial parental assent followed by full consent once the child was stabilised and the situation less stressful (this was later introduced in response, as described below).

The consent information that most concerned parents was blood sampling, specifically concerns about pain and dangerous volumes for sick babies. As one mother explained:

I was worried that with the amount of blood they would drain the child yet she doesn’t have enough of it… Mother.Hh.7. Rural (ref 41, p11).

Frontline staff tried to reassure mothers and explain that many samples collected for CHAIN are primarily for the child’s clinical care not research. These reassurances often stemmed from staff members’ recognition that, as an observational study, there were few biomedical risks associated with participation and potentially important benefits, including close attention during admission and regular funded follow-up clinic appointments. An associated challenge was that when children later deteriorated or even died, the study or staff could be blamed (box 1, quotes 2 and 3).

### Conducting study procedures: emotions, compensation and ancillary care

Staff often raised concerns about levels and types of compensation and benefits that could or should be given to participants’ families, with questions focused on what is ‘allowed’ under the CHAIN protocol and wider institutional rules and whether exceptions can be made for unusual or unexpected cases.

An example of an apparently simple issue raised regularly in the Kilifi CHAIN study site was that different levels of compensation were being given for a research visit to the hospital for similar children in different studies (300/=vs 350/=; US$2.69 vs 3.14). This was because those studies had been approved at different times and institutional policy had shifted a little in line with national minimal pay level over time and because specific figures are required by the national ethics committee. Parents discussed with others in different studies and were clearly unhappy about these differences and frontline staff found it difficult to justify and explain them. Staff wondered why these could not be standardised.

A more complex issue raised by many frontline staff surrounded the emotional issues raised by getting to know the families over the course of the admission and during administration of the socioeconomic, nutrition and mental health questions (Patient Health Questionnaire (PHQ)-9) aimed at ascertaining the child’s household situation. Staff described being previously unaware of those issues in patients’ or participants’ lives and also a sense of regret and guilt about asking questions that raised issues they could not assist with. Issues raising guilt and regret included hunger and lack of money (box 1, quotes 4 and 5) and a recognition that even if the PHQ-9 responses did not indicate referral, some mothers clearly still needed help (box 1, quote 6).

Discussing these issues across CHAIN, several experienced frontline clinician researchers confirmed that the questions were not only enormously challenging to ask but also that responses increased their understanding of and empathy for the needs and vulnerabilities of the families they serve. The CHAIN team also discussed that it would be difficult to assist with these issues in terms of policies and guidance if they remain out of sight. Nevertheless, as described above, it left them with dilemmas regarding if and how to meaningfully assist with very limited resources. As we documented in ethics reflection notes:

- Participants who scored badly on the depression score—how far should we intervene beyond directing them to ‘counsellors’? Will they even go to the counsellors? How busy are our counsellors and will they really be able to follow up?’ (Kilifi ethics reflection meeting 3).
- Social worker is referred to a lot to (potentially) support with a range of livelihood problems—but how busy and able to follow up? Only one in the entire hospital. (Kilifi ethics reflection meeting 3).

In some ways, revealing or hearing about physical health problems among mothers or family members as well as child participants was more familiar for frontline clinical staff and routine referral processes were in place. However, staff worried on one hand that parents would not be able to or wish to follow-up on referrals (out of cost concerns or a different understanding about illness causation), and on the other hand that they should not undermine the study’s social value by interfering with the ‘standard of care’ that CHAIN was observing:

- Participant referred for expensive tests/procedures and can’t afford. CHAIN does not cover these costs. You still require to collect information from this participant on follow up. How do you ignore their plight and proceed with data collection and SAMPLES!!! (Kilifi ethics reflection meeting 2).

A particular concern raised early on in our sessions by a number of fieldworkers was their concerns about having to conduct verbal autopsy (VA) interviews with family members of CHAIN children who died; they felt anxious and underprepared to conduct such emotionally charged interviews despite being highly experienced interviewers from the local community.

### Ending the study

Most participants’ family members were keen to participate and stay in the study in order to access the perceived benefits, even where they had some initial or lingering anxieties or concerns. When the study ended some were disappointed that their children would no longer attend regular research follow-up clinics after the fixed 6-month postdischarge follow-up period and others were relieved, interpreting that it must mean their child had ‘recovered’.

For frontline research staff, corresponding dilemmas were their felt responsibility to continue helping families with clinical needs (described further below) and for how long. One clinician shared that he had been contacted by a child’s parent many months later, illustrating how difficult it can sometimes be for staff and parents to end relationships:

- The other day I was called at home, over the weekend by a participant who was in CHAIN … he wants me to discuss their child, how the child is doing (colleague: Because you have built rapport with them…) yeah…we discussed and then I asked them to kindly come to the hospital he will be helped but its… somebody calls you at night…! (Kilifi ethics reflection meeting 4).

Unfortunately, and as would be anticipated with a study involving very sick children with mortality as the main outcome indicator, some children died. As quotes 2 and 3 in box 1 suggest, the death of a child in a study is hugely emotional for staff, especially when they were held in some way held responsible. Several clinical research staff raised that it felt inappropriate to share emotions about deaths with children’s family members and even with colleagues, mentioning that emotions surrounding death just had to be dealt with ‘in our own way’ and outside work.

### Difficulties in disentangling the personal and the professional

A cross-cutting dilemma raised was if and how frontline research staff could help families personally, where study or institutional processes could not provide support. Many staff did reach into their own pockets to assist, often discreetly (box 1, quotes 3 and 7). In so doing, staff would often mention that they ‘confess’ or ‘admit’ to helping. One fieldworker explained:

- (I told the mother), ‘KEMRI does not allow me to give out money so I haven’t given you this money as a KEMRI staff, it’s like an offering, like the way you give out offerings in a mosque that’s how I am giving you’. So that it gets clearly in her head that it’s not from KEMRI but just a blessing. (FW interview 1, Kilifi)

Clearly emerging across interviews and discussions with clinical staff, particularly those living within nearby communities, was the difficulty of disentangling research and routine clinical care dilemmas, as well as their responsibilities as researchers, clinicians and community members. One clinician described the complexity of this, highlighting that any failures to assist risked being shared with his/her own family:

I don’t have to lie I gave it (the mum some money). This mother has stayed in the ward for three, three/four days (and could not afford to get home) and my other thinking’s like—now think like the medical perspective—this child gets any acquired infection this is going to be a bit more trouble than my two hundred shillings ($2)… then the other bit is like …when they go home you know what they say? …’ I didn’t have fare to come home, they (names the clinician) didn’t do anything’…so that even puts me more at (interviewer: Vulnerable?) vulnerable. (clinician interview 2, Kilifi)

Often, dilemmas raised were largely clinical, with the research context being of minor, if any, relevance. For example, in one meeting there was a concern about a child who the father insisted should be discharged early, despite the clinicians’ (and mother’s) concern about his condition. The clinicians worried that the child would not survive for long without further treatment, but the mother ultimately followed the father’s wishes and took the child home. The clinicians were concerned about their clinical responsibility to the child and whether the police or social worker should be contacted to intervene. A far less prominent concern, only raised on prompting, was whether research(ers) would be held responsible for the anticipated poor outcome or for inappropriately intervening if any action was taken.

Another issue related to concerns about poor outcomes was where local staff wondered if they could or should attend funerals of CHAIN participants if they unfortunately passed away during or soon after the study period. These staff had built relationships with families across multiple home visits. They felt that attending funerals would show respect and provide an opportunity to offer condolences, possibly even in the form of a small financial contribution, as is common practice in the local community. However, they were also concerned that such visits would lead to research staff or the broader institution being blamed for the child’s death, even in the case of a purely observational study like CHAIN. They also worried that such visits may raise expectations and concerns about fairness among families who are not visited by institution staff in this way when they lose children, either because their children were in other studies or not in any studies at all.

### Identifying responsibilities and action in ethics reflection sessions

Ethics reflection sessions would often begin with a discussion of what happened, what worked well and potential challenges with different responses. Beginning with the prompts at the bottom of the ethics reflection guide (figure 2), we would consider the urgency (requires a timely action) and seriousness (in terms of the implications for the person or people involved) of the problem or issue and how related the problem was to the research (was it caused by the research, revealed by the research or related a background situation). We would discuss how much capacity there is for the person to be helped, whether the person trying to help has potential to be harmed and who realistically is best placed to try to assist where assistance is warranted. Illustrations of how dilemmas were discussed are shown in box 2.

Box 2 **Illustrations of how dilemmas we discussed were categorised**

**Level of urgency and seriousness:**

- Urgent: frontline staff members’ concerns about conducting verbal autopsy (VA) interviews.
- Serious: mothers reporting suicidal feelings in PHQ-9 questions; reporting physical violence in homes.
- Less urgent or serious: parents refusing to join the study because of blood samples.

**Relatedness to research:**

- Would not have arisen at all if it was not for the study design or its’ implementation—emotional impact of being asked to answer VA questions; differences in compensation levels between similar patients involved in different studies.
- Increased by procedures or even by simply explaining those procedures—family members’ anxiety on hearing about and seeing volumes of blood sampled from an ill child, even where these volumes are considered biomedically safe and primarily taken to support clinical care.
- Revealed by the study procedures, but not necessarily caused or increased by those procedures - For example, the study SES questions revealed to clinicians the levels of vulnerability in homes among children and parents that they were otherwise not having to directly engage with and the clinical check-ups suggested the need for referrals that were outside those that could be covered by the family, study team or wider institution.

Across the five ethics reflection sessions in Kilifi and the three in Nairobi, all of the issues described in the previous section, and more, were raised and discussed. There was some initial hesitation observed for some people in raising issues in meetings, and some more senior staff needed support in responding constructively to concerns being raised. However overall, there was very positive engagement in the ethics reflection sessions, with many participants sharing that they were grateful to have an opportunity to discuss their worries and dilemmas with peers and bosses; some mentioned they had not had such an opportunity in previous clinical studies.

- (as clinicians) we are really programmed to only ask about what we can fix… we are just trained into service provision and the issues of making things better. So, in terms of asking the questions then we can’t fix things, it feels very uncomfortable for us, and I think for the reflection that’s why this is useful (ethics reflection meeting).

Discussions of the issues raised included important and often quite complex debates on how to get the balance right between giving adequate support and benefits and avoiding (undue) inducement and between assisting an individual family and introducing unfairness between families or undermining the study design. We drew on previous community consultations in our setting highlighting that concerns about undue inducement in low-income communities can often be misplaced and that there should be greater attention in practice to avoiding unfair levels of support, particularly for the poorest families.^45^ There were debates on whether actions would set precedents and have implications for other studies and the health system, what those implications would be and how to ensure any interventions were as sustainable and meaningful as possible.

Actions of some form were almost always needed and possible and in some cases were relatively clear. For example, an assent process was introduced through a change in the study protocol approved at institutional and national levels (level C action). The study ancillary care plans, including contact details for different scenarios, were discussed, clarified and expanded and support processes for staff handling patient deaths, regardless of the child’s involvement in research, were reviewed and strengthened.

Actions were often required across several levels in figure 2 (levels A–E). An example was fieldworkers’ concerns about conducting VAs for children who died at home after discharge from hospital. We agreed this issue was urgent and would not have arisen at all if it were not for the research. Although those expected to conduct VAs appreciated that they were able to raise and share their concerns with line managers and colleagues (level A), action was also needed, including:

- Level B—sharing ideas—Sharing ideas to minimise the emotional burdens on selves and parents, including active listening, demonstrating patience and agreeing on interview timing and respecting cultural and religious norms around burials. Also, organising further specific training and advice sessions from more experienced VA interviewers.
- Level C—study—During the above training, a potential risk that parents might unintentionally be subjected to two VA interviews by different research teams was identified. Co-ordination across studies was increased to prevent this.
- Level C—institution—We organised wider institutional discussions on: when VAs can be justified scientifically; the emotional and moral dilemmas involved for families and frontline staff and how to appropriately support frontline staff in conducting these interviews. These discussions underscored the importance of pre-existing institutional policies that minimise the inclusion of community members in multiple studies involving invasive procedures.

Given the range of vulnerabilities faced by so many families,^31^ inevitably there were many issues that individual staff and the wider team could not assist with. For example, in terms of the public facilities or services to which referrals were made, the CHAIN team recognised and remained concerned about the existing resources, quality and follow-up. Also, where action was recommended that was out of the study team’s control, this involved many different people and took time; as a result, some agreed actions were rejected or seen as impossible or impracticable at the institutional or health system level. Those running the ethics reflection sessions needed to continuously communicate, follow-up and share feedback on actions taken and next steps in order to maintain trust, energy and interest in the sessions.

Overall, frontline staff described a psychological relief in sharing their experiences and concerns, as well as valuing rapid responses and agreement around practical solutions. A senior staff member highlighted that the sessions had emphasised to them the importance of supporting frontline staff not only in building relationships with participants and family members but also in working out how to end a relationship when the study ends, participants withdrew or there is a death (made even harder by the careful relationship building):

- Personally I emphasise in my training on consenting in research especially for frontline staff, (the need) to build rapport and… to build a relationship, what I never get to train them… it’s never even crossed my mind, is how to get out of it. (ethics reflection)

One of the senior clinical study PIs who has led many previous large-scale studies and trials mentioned that these types of sessions should be incorporated into every study, ‘not just for the ethics but also for the science and the quality of the data. It should just be routine practice in studies’.

## Discussion

In our setting, frontline research staff—both clinical and non-clinical—faced a wide range of ethical issues and dilemmas in their interactions with research participants and family members. These dilemmas emerged from: (a) the intrapersonal, interpersonal, environmental and structural vulnerabilities that family members’ faced,^31^ (b) the efforts that family members made to access care and support for their children and other family members in these contexts^31^ and (c) staff members’ felt obligations and real or perceived constraints to intervening, given the study design and agreed SOPs, the research institution’s policies and processes and the wider health system context (described in detail elsewhere^46 47^).

The ethics reflection tools and approach we developed were not planned in advance, but evolved from a practical need for support from colleagues. They built on our past experience of conducting participatory training with fieldworkers^26 34^ and in particular in running ethics-focused team debriefing sessions for health policy and systems research (HPSR).^43 48 49^ The latter in turn arose from a relatively routine practice in qualitative research of conducting debriefs, where team members meet post fieldwork to discuss the ‘tenor, flow and findings’ of a research activity.^50^ Debriefs can be a discrete and essential supplement to qualitative methods with the notes making an important component of the full data set. Incorporating systematic debriefs can help build researcher capacity, support data quality, allow the study to evolve in line with contextual issues and emerging insights and support sharing of emerging findings with collaborators.^50^ Debriefs can also contribute to broader reflexivity, whereby researchers critically reflect on the way in which they construct knowledge; the sorts of factors that influence their research topics and focus and the planning, conduct, analysis and writing up of the research.^22^

Ethics issues are not generally a focus in qualitative research debriefs or reflective practice.^22 50^ However, in our past HPSR debriefing sessions, we specifically sought to unpack issues where there was moral discomfort or uncertainty and later related the discussions, decisions and emerging dilemmas back to ethics principles and guidance.^48 49^ For the CHAIN-related sessions shared in this paper, we took this a step further by drawing on the benefit-sharing and ancillary care literature and in particular on Richardson and Belsky’s partial entrustment model,^44^ to guide the discussions themselves. The questions indicated in figure 2 helped us unpack each ethical issue, or ‘moment’, to consider our own and others’ responsibilities. Context was taken into account through drawing on participants’ differing expertise and experience, including a deep understanding of local cultures, norms and sensitivities (frontline staff from the area), tacit knowledge of healthcare and referral services (clinical team members who have provided clinical care for years) and an inside understanding of institutional and broader research processes and oversight, including community engagement and empirical ethics research (clinician and social science researchers and an external bioethicist). We constantly referenced in our discussions (and sought to amend where necessary) the study ancillary care plans and informed consent SOPs, as well as the institutional consent and benefit-sharing policies that informed them.^16 34 45^

In incorporating questions from ethics literature and guidance into our sessions, it could be argued that we developed an approach to operationalise the ethical mindfulness or reflexivity recommended by Guillemin and Gillam^22^ for social scientists and applied it to an interdisciplinary clinical observation study. Given the nature of the study and its' hospital base, the sessions we evolved and issues raised resonate with reflective learning approaches used in medical teaching (which involve critical reflection about economic and power relationships and reflexivity about one’s own assumptions and behaviours^51^) and forms of ethical support services developed to better handle ethical challenges in healthcare.^52 53^ Regarding reflective learning approaches, there was mutual learning about how to handle and respond to similar situations as we moved forward in conducting this and similar studies. Regarding ethics support sessions, we combined elements of ethics reflection groups (ERGs) or moral case deliberations, where an interdisciplinary group of clinicians reflect on a specific ethical challenge from everyday clinical life, with elements of ‘clinical ethics committees’ or ‘ethics consultations’ which operate more at hospital level.^52^

A limitation of our approach is that we did not formally evaluate it. Nevertheless, we did receive valuable feedback and learnt important lessons. An important early lesson was the need to build the levels of trust between frontline staff, social scientists and CHAIN senior researchers in order to normalise the process of sharing ethics concerns. Research processes in many settings inevitably interact with existing societal relations of power at many levels, including between different members of research teams (international and national, biomedical and social science, clinical and non-clinical, facility based and field based).^27^ In our case, there was an initial impression that the ethics and social science team were acting as the ‘ethics police’ and that sharing issues might get staff into trouble, an issue that has been observed in embedded ethics work in resource-rich settings.^54^ Beginning with informal conversations, gradually adding ethics discussions to team meetings to normalise practice and leading by example (having social science researchers and more senior staff volunteer issues) appeared to build trust over time. The sessions began to highlight the complex roles that frontline staff play and how challenging it can be to negotiate even the apparently ‘simple’ issues they confront daily. They became an opportunity to build supportive relationships across hierarchical research teams and to support one another to navigate complex power-related issues and intersecting vulnerabilities among participants/community members.

Although some of the issues raised in our sessions were very specific to the clinical observation study being conducted, many resonated with those reported from a wide range of empirical ethics studies conducted in the region,^11 19 55^ suggesting the potential relevance of the approach beyond the specific study and study team and its institutional or geographical context. In many settings, health research team members will be juggling research and healthcare responsibilities and struggling to cope in what can be structurally and emotionally extremely challenging contexts,^56^ now significantly exacerbated by the stresses associated with the COVID-19 pandemic.^57^ As noted by Bruun *et al*^52^ with ERGs, our sessions did not always result in unequivocal solutions to the ethical challenges faced, but they did appear to offer an important possibility to share perspectives and decisions on challenges faced and how to manage them, with the potential to reduce the significant moral distress associated with keeping them private or pushing them aside. Discussing study ancillary care plans and consent SOPs helped to bring these documents to life for frontline staff and generate ideas about best practice for implementation, as well as highlight areas where these documents could be updated and strengthened.

It is important to acknowledge the investment required in making such an integrated ethics approach to research possible. This was a funded embedded ethics study in an institution with strong research ethics capacity within existing staffing. Even so, the time commitment was substantial and facilitation required training and expertise in managing ethics reflections. Strong support and engagement from the senior clinical study leads was essential for the entire process but even then, several meetings did not have full attendance, where other pressing deadlines came up or where there was no clinical back up to deal with needs of participants.

Finally, and crucially, the specific tools and approach we developed should complement and feed into—and certainly not be instead of—an ethical study design with agreed social value, as well as carefully developed and implemented institutional ethics review, safeguarding and health and safety policies and wider staff training and career support initiatives. Ethics reflection sessions and associated activities will likely raise issues that overlap with and potentially require referral to these broader policies (as outlined in our case in figure 2). For example, a child’s father being angry with a fieldworker and threatening him or her with violence may be both an ethical issue and a staff health and safety concern. A mother reporting domestic abuse or a child being discharged from hospital against medical advice could also be considered a safeguarding issue. A staff member being unable to raise an issue with a supervisor or feeling uncomfortable may be a safeguarding issue, be linked to bullying and harassment, or reflect challenges in support processes and job security. Emotional issues for staff may point to necessary changes in consent or ancillary care policies or in some cases require referral for individual counselling. We are fortunate that these policies and procedures are in place at KWTRP, but here as elsewhere they are far from straightforward to implement in practice and need continuous review and amendment.

## Conclusion

Health research in low-resource settings will inevitably involve groups defined as ‘vulnerable’ in research ethics guidance. For paediatric clinical research, studies such as CHAIN will often involve children and family members facing multiple layers of vulnerability. We have shared an approach to support frontline staff in identifying, reflecting on and responding to ethical dilemmas throughout approved studies. Numerous dilemmas were raised and shared. Sometimes responses and actions were relatively straightforward to agree and implement, but often they were not. Overall, the practical and emotional realities and challenges of working in contexts of multiple layered vulnerabilities, including structural drivers largely beyond the control of individuals and the study, were highlighted.

We suggest that such approaches are incorporated into all health studies in low-resource settings (including clinical observation studies, trials and HPSR) and that they feed into—where appropriate—changes to the study SOPs and protocols. This requires institutional and national policies and practices that recognise the importance of being responsive to emerging ethical issues in research. Importantly, the studies themselves should be designed and regularly reviewed for their potential to contribute to positive transformation locally and more widely. Ethics support processes post approval require sustained relationship-building work overtime, not least given potential concerns among frontline staff about fault-finding and blame in contexts of strong research institutional hierarchies. The latter can arguably help ensure studies addressing the needs of participants and families are successfully implemented in complex institutional, socio-cultural and political contexts, but they can also prevent important community and frontline issues from being raised and shared. Such initiatives also require the right expertise and proactive senior support to ensure discussions are non-blaming and constructive,^58 59^ and strong collaborative relationships with health managers and leaders of NGOs to support the development and implementation of locally appropriate ancillary care and referral plans.

We intend to try out different types of ethics reflection discussions in different contexts to explore if and how they work and what they need to function effectively over time (in terms of supporting frontline staff and responding to participant vulnerabilities). We would value inputs and ideas from others and welcome further evaluation of our shared tools and approach.

## Data Availability

Data may be obtained from a third party and are not publicly available. The datasets generated and analysed during the study are not publicly available due to institutional rules and regulations but may be available from the corresponding author on reasonable request.
