# Supplementary material for: Model for developing context-sensitive responses to vulnerability in research: managing ethical dilemmas faced by frontline research staff in Kenya
Source: BMJ Glob Health. 2021 Jul 8;6(7):e004937. doi: 10.1136/bmjgh-2021-004937 (PMC8268889; doi:10.1136/bmjgh-2021-004937)
Supplement: Supplementary data [file bmjgh-2021-004937supp001.pdf]

Supplementary file 1: Vulnerabilities and constrained agency revealed in family treatment-seeking stories and encounters with research

<table>
<tr><th></th><th>Vulnerabilities</th><th>Indications of agency</th></tr>
<tr><td>In the home OR COMMUNITY</td><td>Intrapersonal
- Carers’ poor physical and mental health and well-being
- Anxieties about child health and well-being, and about elements of the research

Interpersonal - Complex family situations & dynamics
- Family members living in split households
- Carers’ limited access to or control over income
- Changes in living arrangements
- Carers’ facing psychological or physical abuse – including potentially blame for agreeing to or refusing their child’s agreement in research

Environmental – socio-economic & cultural
- Low access to funds and competing demands for funds, including small research related reimbursements
- Symptoms perceived as normal or to require treatment from healers</td><td rowspan="2">Intrapersonal
Mothers’ actions to help their child:
- Visiting many facilities
- Shifting, repeating and mixing sources of care
- Accessing care on credit

Interpersonal - Accessing support from others in the home and community (including research related elements)
- Seeking out and acting on advice
- Securing funds (& loans)
- Working with others to persuade husbands
- Negotiating for delays in payments
- Seeking and giving support to others

Environmental – socio-economic and cultural, and institutional
- Reorganising living arrangements
- Rethinking feeding arrangements
- Changing work practices
- Demanding information and support from health providers, cleaners, security guards and others in health facilities

Research specific
- Refusing the blood sampling element of research participation
- Choosing to consent to the study and stay in it despite rumours being raised and shared by other mothers in the wards, and others in their homes and communites</td></tr>
<tr><td>Interactions with facilities, research(ers) and broader health systems</td><td>Intrapersonal
- Emotional and practical concerns about child/ren and about quality and costs of care; sometimes exacerbated and sometimes eased by (because asked about) research participation

Interpersonal - Power relations between staff & parents
- Being treated with disrespect
- Fear to ask questions, share info or demand attention
- Lack of trust in care and advice given
- Anxieties about whether will get research results

Environmental – socio-econ, cultural & institutional
- Cost or distance from desired facilities
- Avoiding facilities considered poor quality or costly
- Unclear or conflicting referral and continuity of care</td></tr>
</table>
