# Supplementary material for: Model for developing context-sensitive responses to vulnerability in research: managing ethical dilemmas faced by frontline research staff in Kenya
Source: BMJ Glob Health. 2021 Jul 8;6(7):e004937. doi: 10.1136/bmjgh-2021-004937 (PMC8268889; doi:10.1136/bmjgh-2021-004937)
Supplement: Supplementary data [file bmjgh-2021-004937supp002.pdf]

Supplementary file 2 – illustrations of family background situations and treatment-seeking journeys

Illustrations of background family situations

Hamisi: A child aged 1 year 9 months on admission (RURAL): This child lives in an extended rural home with four siblings, his 20-year-old mother, and the child's paternal uncle and grandmother. His mum has no formal education or income. She relies on farming and her husband's income, with some help from the grandmother. The father lives and works in another rural location on rented land; visiting home every 2 months or so, as funds allow.

Khadija: A child aged 14 months on admission (RURAL): This child lives with one older sibling and her mum in their rural home, with the father's relatives. Two other siblings (aged 8 and 4) live in Mombasa with their maternal grandmother. The child's mother has no formal education and does casual work for a living (cultivating others' land, fetching water, pounding maize). She used to burn and sell charcoal but gave up because her husband would take all her money. The child's father is a motorbike driver and helps deliver palm wine to his father's bar. He attempted to commit suicide during our study period after an argument over KShs. 600 [$ 6] he had been given by his father for his daughter's school fees.

Mercy - Child aged 1 year 5 months (URBAN): This child is aged 1 year, 5 months and lives with her 22-year-old mother, her father, and three older siblings. Her mother is the youngest of 4 wives (two other wives are deceased), having been 'inherited' by her late sister's husband. Mercy's mother has primary level formal education and is unemployed, although sometimes she does casual jobs washing clothes. She primarily relies on her husband – who also works as a casual labourer - for financial provision; and sometimes gets help from her own mother who lives in the village.

Illustrations of treatment-seeking journeys prior to admission

Baraka: Aged 13 months on admission (Kilifi County URBAN). Baraka's mum is a college educated nursery teacher, and Baraka had been sickly and poor feeder from birth. The mum had sought treatment from a series of places in the months leading up to admission including the local shop, two different public health care facilities (at the second she was hugely hurt when accused in public of failing to take proper care of her child, a healer (on neighbours' advice – 'as a parent you will try anything'), and finally another government facility where the child was referred for admission at Kilifi County Hospital.

Hamisi: Aged 1 year 9 months on admission (RURAL): Hamisi started losing his appetite, vomiting and experiencing diarrhoea several months before admission. His mum and grandmother thought his 'kilimi'- uvula – was causing the vomiting. She took him to the father's place of work and he organised for it to be cut out by a healer. However, symptoms persisted for a further month so she took the child to a series of healers (3) who diagnosed possession by evil spirits and recommended treatment. But Hamisi began to swell and so the mum consulted a retired health worker in the village who told her the child's blood was low and prescribed medication. This did not work and another retiree in a neighbouring village diagnosed kwashiorkor and administered an injection. A neighbour advised her to go to a government dispensary and convinced her own husband to buy a cockrel from the mother so she had money to get to the facility. On the second visit to that facility, she was referred to another government hospital in a nearby town. On consultation with the husband and following advice from neighbours, she decided instead to go to Kilifi hospital which is closer and more familiar (the father had to be persuaded against going to a healer). By the time of admission she'd already spent over KShs. 2300 [$ 23] on treatment and unpaid balance at one of the private practitioners.
